# Optimization of Induction Protocols for Experimental Autoimmune Myasthenia Gravis

**DOI:** 10.3390/ijms26104628

**Published:** 2025-05-12

**Authors:** Xiangrui Zhang, Yu Bai, Shida Wang, Jun Shi, Haoxin Wu

**Affiliations:** 1 Key Laboratory of Integrative Biomedicine for Brain Diseases, School of Chinese Medicine, Nanjing University of Chinese Medicine, Nanjing 210023, China; 202430001@njucm.edu.cn (X.Z.); 20210002@njucm.edu.cn (Y.B.); 20220003@njucm.edu.cn (S.W.); 2 National Famous Chinese Medicine Expert Inheritance Studio (Meng Jingchun), School of Chinese Medicine, Nanjing University of Chinese Medicine, Nanjing 210023, China

**Keywords:** myasthenia gravis, experimental autoimmune myasthenia gravis, optimized model induction protocol

## Abstract

Myasthenia gravis (MG) is an autoimmune di sease characterized by muscle weakness. Experimental autoimmune myasthenia gravis (EAMG) serves as an animal model for MG research. Despite advancements in EAMG modeling, limited drug absorption and variability in disease manifestation among animals resulted in a low success rate of model induction. This study aimed to optimize and standardize the modeling process by exploring different induction conditions to improve success rates. We employed female Lewis rats and C57BL/6 mice to compare their sensitivity to model induction and applied a controlled variable approach to acetylcholine receptor (AChR) and H37Ra dosage, mixing time, and injection sites. Results showed that Lewis rats were more suitable than C57BL/6 mice, and 75 µg AChR peptides were more effective than 50 µg. The immune-boosting effect of 1 mg H37Ra Mycobacterium tuberculosis was weaker than 2 mg. While drug mixing time had little impact, increasing injection sites on backs and including foot pads injection, significantly improved drug absorption.

## 1. Introduction

Acquired myasthenia gravis (MG) is an autoimmune disease primarily characterized by muscle weakness or easy fatigability, which cannot be relieved by rest [1]. The pathogenesis of MG is complex, and it is often attributed to the impairment of neuromuscular junction (NMJ) transmission caused by specific autoantibodies targeting the acetylcholine receptor (AChR) on the postsynaptic membrane of skeletal muscle cells [2]. In recent years, the incidence of MG has increased, particularly among the elderly.

The history of animal models for MG dates back over 50 years [3]. Initially, MG was induced in rabbits using AChR purified from the electric eel, which led to MG-like symptoms in these animals. Later studies confirmed that anti-AChR antibodies were responsible for the autoimmune response in both MG patients and animals, causing structural and functional abnormalities at the NMJ [4]. Over time, experimental autoimmune myasthenia gravis (EAMG) has become a valuable model for investigating the pathogenic mechanisms of human MG and for evaluating new immunotherapies [5].

Although EAMG serves as a useful model, there are notable differences between clinical MG in humans and animal models. A significant difference is that MG patients often exhibit thymic alterations, which are absent in EAMG animals. Therefore, EAMG may not be ideal for studying thymus involvement in MG [6]. However, many similarities exist between MG patients and EAMG animals, including muscle weakness, fatigability, decreased response to repetitive nerve stimulation, and temporary recovery of muscle strength following treatment with anticholinesterase drugs.

MG can be induced in various animal species, but rats and mice are most commonly used due to the high incidence of clinical EAMG symptoms, their easy availability, and low cost [7]. During model induction, muscle strength and body weight loss are monitored in immunized animals. Myasthenic symptoms, such as tremor, hunched posture, muscle weakness, and fatigue, are assessed after exercise. Other key indicators for evaluating the model include reduced muscle endurance, increased serum inflammatory factors, and T cell subset imbalances in immune organs.

Animal models of MG can be broadly classified into several types. In susceptible rat strains, EAMG is typically induced through active immunization with either Torpedo californica AChR (TAChR) [7] or with a rat AChR epitope (amino acids 97–116 of the α-subunit) [8], which can break immunological tolerance [9]. Alternatively, EAMG can be induced via passive transfer of anti-AChR antibodies, which is the simplest method for studying the pathogenic effects of autoantibodies in vivo.

- Active EAMG

EAMG model of mice

EAMG has been extensively characterized in murine models to elucidate pathogenic mechanisms and evaluate therapeutic modulation. Susceptibility studies identify C57BL/6, SJL, and AKR strains as particularly vulnerable [10], with 50–70% of immunized animals developing measurable neuromuscular deficits following Torpedo californica acetylcholine receptor (TAChR) administration [11]. The selection of this animal model was not only reported in early studies following the establishment of EAMG but has also been consistently adopted in recent related research [12]. Standardized protocols involve primary immunization with 20 μg purified TAChR emulsified in complete Freund’s adjuvant (CFA), succeeded by two or three booster doses in incomplete Freund’s adjuvant (IFA) at 20 μg concentration. This regimen induces cross-reactive antibodies targeting both xenogeneic TAChR and endogenous murine AChR, with clinical manifestations typically manifesting 7–14 days post-final immunization.

TAChR induced EAMG

The Lewis rat strain has emerged as a predominant model for active EAMG induction, demonstrating enhanced disease severity compared to murine counterparts. Immunization protocols using TAChR in Lewis rats usually achieve 80–90% disease incidence through single-dose administration (20 or 30 μg in CFA), circumventing the multi-boost requirements of murine models [13]. This system generates cross-reactive antibodies against both xenogeneic and endogenous rat AChR with 3.1-fold higher mean titers than mouse models [11]. Notably, the Lewis rat model recapitulates two distinct disease phases observed in human MG pathogenesis, with the first acute phase (Days 7–14) characterized by the IgM type of anti-AChR antibodies, which leads to complement depositions on muscle membrane, extensive phagocytic invasion at the NMJ, and destruction of the postsynaptic membrane [14]. The second progressive chronic phase (Days 21–60) is characterized by the production of a larger amount of IgG type antibodies and complement deposition at the postsynaptic membrane and NMJ destruction [7], which thus appears flat due to lack in junctional folds.

Synthetic peptide induced EAMG

In rats, active EAMG can be induced through immunization with a synthetic peptide corresponding to the immunogenic region 97–116 of the rat AChR α-subunit (R97–116), administered in CFA at a dose of 50 mg. A second immunization with R97–116 (50 mg) in incomplete Freund’s adjuvant (IFA) is performed 30 days after the initial injection. EAMG symptoms typically manifest approximately 10 days following the booster immunization [8].

There are notable differences between EAMG induced by the rat AChR peptide (R97–116) and by the TAChR, particularly in terms of the disease progression. R97–116-induced EAMG is characterized by a slower progression and greater clinical variability among immunized animals [15]. Due to the superior feasibility of using the synthetic rat AChR peptide compared to the TAChR, most studies on the etiology, pathogenesis, and therapeutic strategies for EAMG have utilized the R97–116 experimental model.

- Passive transfer of EAMG

EAMG can also be induced through the passive transfer of autoantibodies, which can be achieved using two main approaches. One method involves the injection of purified serum IgG, isolated from myasthenia gravis (MG) patients, or anti-AChR antibodies purified from AChR-immunized chronic EAMG animals into healthy recipients [16]. Additionally, passive EAMG can be induced by administering monoclonal antibodies (IgG1 or IgG2a) targeting the AChR α-subunit. These antibodies are typically derived from AChR-immunized animals or from cell culture supernatants. This protocol triggers EAMG symptoms in recipient animals within 24 h. This passive transfer model serves as an excellent tool for characterizing the immunopathogenesis of AChR-MG, investigating the pathogenicity of other autoantibodies targeting different antigens [17], and evaluating the therapeutic potential of drugs specifically aimed at mitigating the effects of auto-antibodies.

- Adoptive transfer of EAMG

Beyond the passive and active induction methods mentioned above, EAMG can also be induced by adoptive transfer. In this approach, human tissues and cells are transplanted into severe combined immunodeficiency (SCID) mice, which lack mature T and B cells and are tolerant to xenotransplantation [18]. Studies have shown that SCID mice engrafted with thymus tissue fragments from MG patients can produce human anti-mouse AChR antibodies within 1–2 weeks post-transplantation. These findings highlight the critical role of the thymus in MG pathology, as it contains the necessary cellular components for producing autoantibodies that trigger the disease [19]. Similarly, SCID mice injected with peripheral blood lymphocytes from MG patients exhibit typical clinical manifestations of MG, including elevated circulating anti-AChR antibodies and IgG deposits at the NMJ. This model further confirms that CD4+ T cells, but not CD8+ T cells, are essential for the pathogenesis of MG.

- Other auto-antibodies in MG and other EAMG models

While AChR antibodies are the primary cause of MG—accounting for approximately 85% of generalized MG and 50% of ocular MG—recent research has demonstrated the existence of AChR-negative MG [20]. In these cases, patients do not produce AChR antibodies but instead may have other autoantibodies that contribute to the disease.

MuSK EAMG

For example, in approximately 40% of MG patients who are negative for anti-AChR antibodies, autoantibodies targeting the postsynaptic muscle-specific tyrosine kinase (MuSK) can be detected [17]. These patients often present with bulbar and facial muscle weakness, along with extreme fatigue, which can be challenging to treat effectively. Experimental animals immunized with MuSK (active MuSK EAMG) develop MuSK-specific autoantibodies and muscle weakness, accompanied by a reduction in postsynaptic AChR numbers, decreased amplitudes of endplate potentials, and impaired neuromuscular transmission [21]. Furthermore, sera from MuSK-immunized mice and supernatants from cultured lymph node cells exhibit elevated levels of interleukin (IL)-4 and IL-10. Similar findings have been observed in mice that received IgG from MG patients positive for MuSK autoantibodies, thereby establishing the passive transfer model of MuSK EAMG [22].

LRP4 EAMG

Furthermore, some cases have identified that in MG patients who are double-negative for both anti-AChR and anti-MuSK antibodies, there is an increased presence of autoantibodies against LRP4, an agrin receptor essential for NMJ formation [23]. LRP4 autoantibodies have been proved to be pathogenic, as demonstrated in mice immunized with the extracellular domain of LRP4, which develop anti-LRP4 antibodies and exhibit MG-like symptoms [24]. Additionally, mouse anti-LRP4 antibodies can inhibit agrin-induced MuSK activation and AChR clustering, suggesting a potential pathophysiological mechanism. Passive transfer experiments have further confirmed the pathogenicity of LRP4 antibodies and established that LRP4, a key player in NMJ maintenance, acts as an autoantigen in MG [25].

As basic research on MG continues to advance, researchers have made several new attempts in modeling the disease. The primary method involves the injection of AChR antibodies or anti-AChR antibodies, often followed by one or two booster immunizations, leading to the development of muscle weakness symptoms in experimental animals [26]. Induction of EAMG typically relies on injecting the AChR or its immunogenic fragments into animals to elicit an autoimmune response similar to that seen in human MG [27]. In addition to vaccinating animals with AChR, various alternative methods have been explored, such as transplanting primary AChR-presenting cells or utilizing genetically modified animals. However, these approaches tend to be more complex and have a lower success rate.

Additionally, pigs and rabbits have also been reported to induce EAMG models, rats and mice remain the most commonly employed experimental animals for EAMG studies. The most frequently used strains include Lewis rats and C57BL/6 mice, with female animals being preferred for model development, as estrogen secretion in females is more conducive to the induction of autoimmune diseases.

In conclusion, although EAMG model induction has been extensively reported, no consensus has been reached regarding a standardized protocol. Substantial variability in species selection, antigen formulation, and immunization regimens has resulted in inconsistent disease severity and immune responses across studies. This lack of consistency complicates reproducibility and limits the comparability of findings, posing challenges for researchers seeking to select or adapt an appropriate model.

To address the current lack of consensus and standardization in EAMG model induction, this study systematically compares key variables across commonly used protocols that reported in previous studies, including species selection and antigen dosage, injection strategy, and adjuvant use. We aim to establish an EAMG induction protocol that balances convenience and cost-effectiveness, utilizing recombinant AChR as an antigen and readily available animal strains, thereby producing a disease phenotype that closely resembles that of clinical patients. Based on these comparisons, we propose an optimized and reproducible induction protocol aimed at improving consistency and translational value in EAMG-related studies.

**Figure 1:**
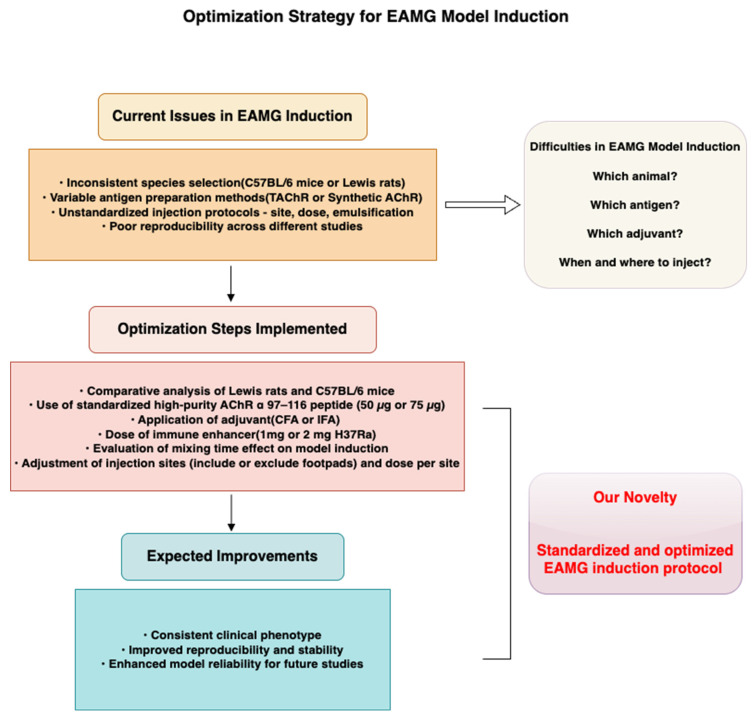
Schematic illustration of the optimization strategy for EAMG model induction. The figure outlines key limitations which contribute to difficulties in successful model induction in existing protocols, the comparative optimization steps implemented in this study, and the expected improvements. It also highlights the novelty of this work—a standardized and optimized EAMG induction protocol aimed at improving reproducibility and stability.

A schematic overview of our approach and proposed workflow is presented in Figure 1, which visually summarizes (1) the comparative design used to evaluate critical induction parameters, and (2) the standardized strategy developed to enhance model reproducibility and stability. This framework helps to clarify the rationale, innovation, and practical significance of our study.

## 2. Results

### 2.1. C57BL/6 Mice Exhibited Limited Susceptibility to EAMG Induction Under Current Conditions

We compared the results in C57BL/6 mice, a strain used in several previous studies, where considerable effects were reported. However, in this study, we found that the C57BL/6 mice strain was not ideal for EAMG modeling.

**Figure 2:**
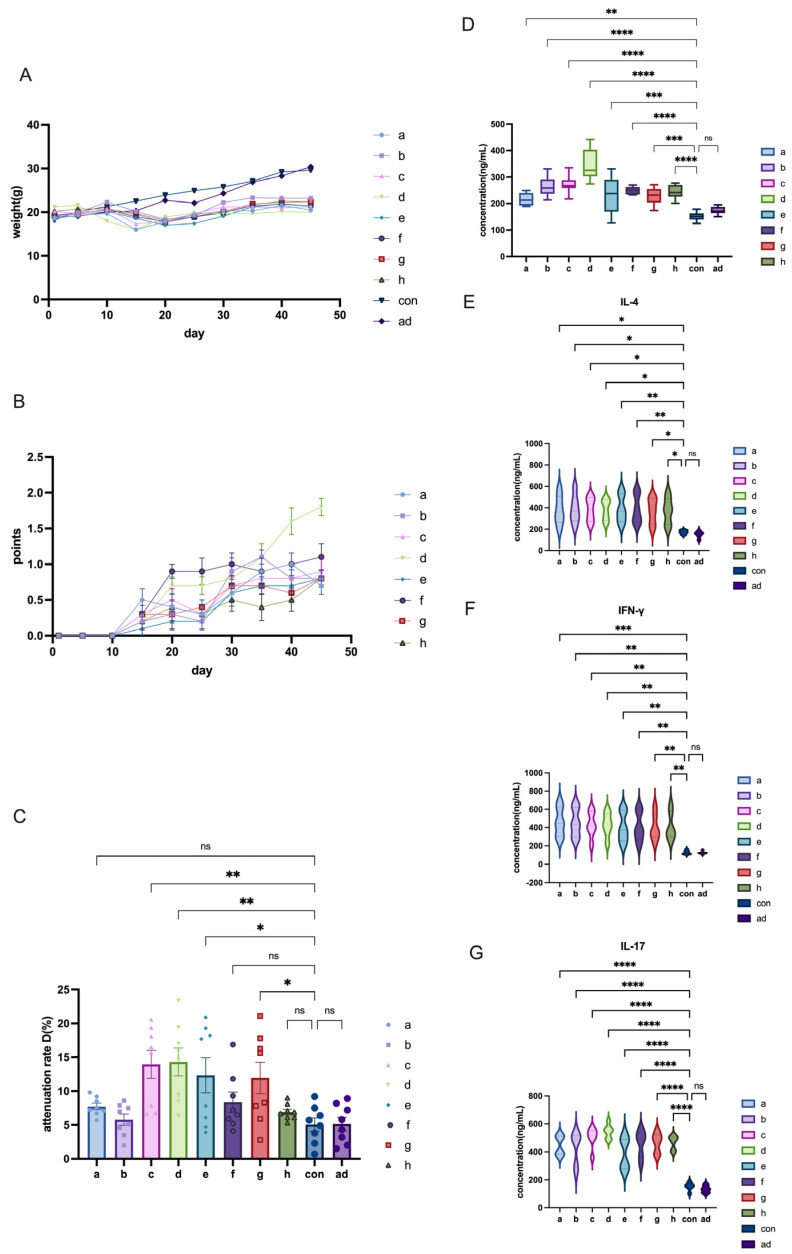
Evaluation of body weight, clinical scores, RNS tests, and ELISA results in C57 mice. (**A**) Body weight changes across different groups. (**B**) Clinical score progression in different groups. (**C**) Attenuation rate D value among various groups in the RNS test. (**D**) AChR concentration across different groups. (**E**) Levels of IL-4 cytokines in C57 mice. (**F**) Levels of IFN-γ cytokines in C57 mice. (**G**) Levels of IL-17 cytokines in C57 mice. All data are expressed as mean ± SEM. One-way ANOVA tests were applied between groups. The data are representative of three independent experiments with consistent results (* *p* < 0.05, ** *p* < 0.01, *** *p* < 0.001, **** *p* < 0.0001, ns: not significant).

We focused on the weight of C57BL/6 mice, a key indicator for the success of EAMG model induction. At the start of the experiment, the weight of all groups was at a similar baseline (Figure 2A). By the end of the induction period, the modeling groups had lower weights compared to the control and adjuvant control groups. This initially seemed to suggest successful model induction. Although a slight trend toward weight loss was observed in the modeling group, no clear difference in body weight was noted between groups at the end of the induction period based on the available sample size (n = 8 per group).

Clinical scores for the C57BL/6 mice were recorded from Day 1 to Day 45. As shown in the figure, symptoms of muscle weakness began to appear on Day 10. From Day 10 to Day 30, the disease progressed steadily, with clinical scores gradually increasing (Figure 2B). However, from Day 30 to Day 45, only Group d showed a continued increase in clinical score, while the scores for the other groups stabilized or even decreased. Notably, even the mice in Group d, which had the highest clinical score, did not exceed a score of 2, failing to meet the typical criteria for EAMG models (Table S1).

Given the limitations of these assessments, we also incorporated RNS and ELISA into the evaluation. In the RNS test, both the control and adjuvant groups showed attenuation values below 10%, indicating no reduction in muscle endurance. The D value for Groups c and d were higher than for the other groups (Figure 2C), but upon examining individual data points, we found that about half of the values did not exceed 10% (Table S2). Therefore, we could not conclusively determine the success of the EAMG model induction. The AChR-ab levels in the model induction groups were higher than those in the control or adjuvant groups, confirming that the induction successfully raised AChR-ab levels (Figure 2D). Serum inflammatory cytokine levels were also measured by ELISA. IL-4, IFN-γ, and IL-17 expression levels in Groups a to h showed upregulated trends (Figure 2E,F), while the control and adjuvant groups exhibited similar concentrations. Notably, the expression of IL-17 in Group d was the highest among all groups. Although there were statistically significant differences in inflammation levels between the model and control groups, the differences were not highly significant.

In conclusion, we were unable to induce a successful EAMG model in C57BL/6 mice in this study. Although AChR-ab levels and pro-inflammatory cytokine levels were elevated, the clinical scores and RNS results did not meet the criteria for a successful EAMG model. Additionally, several practical challenges made this strain less suitable for EAMG modeling. First, the small body size of the mice made it difficult to accurately locate injection sites and perform the RNS test. The small volume of serum collected also made ELISA detection challenging. Furthermore, massaging the injection site after subcutaneous drug administration was difficult, leading to poorly absorbed subcutaneous masses and drug accumulation. Lastly, C57BL/6 mice are known to be aggressive, and mice in the same cage often fought, especially in the later stages of modeling, resulting in poor overall health and making them unsuitable for follow-up experiments.

### 2.2. Better Model Induction Effect in 75 µg AChR 97–116

After determining the appropriate animal species, we proceeded to compare the dosage of peptides, which play a crucial role in model induction.

**Figure 3:**
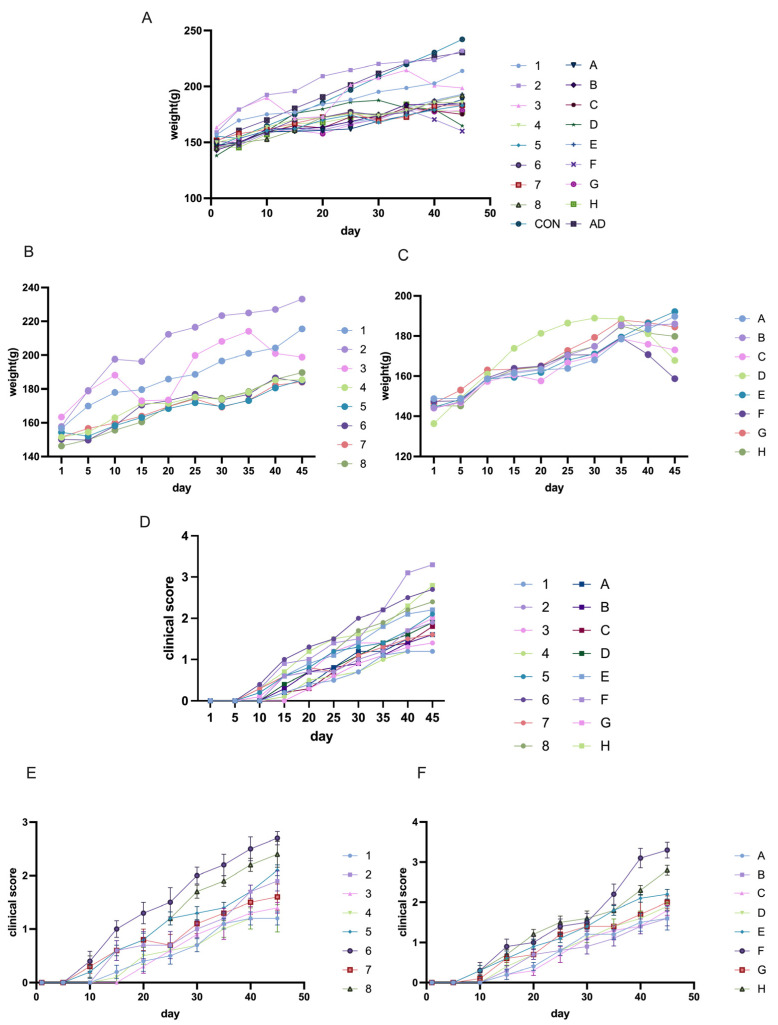
Body weight and clinical score analysis of Lewis rats across different groups. (**A**) Body weight changes in all groups of Lewis rats. (**B**) Body weight curve of the 50 µg groups. (**C**) Body weight curve of the 75 µg groups. (**D**) Clinical score progression in all groups of Lewis rats. (**E**) Clinical score curve of the 50 µg groups. (**F**) Clinical score curve of the 75 µg groups. The data are presented as Median.

In this study, we compared the success rates of model induction between the 50 µg and 75 µg groups. In Lewis rats, we first focused on weight as a key indicator. As shown in the weight curve, the initial weights of all groups were similar, with the control and adjuvant groups showing a steady increase in body weight (Figure 3A). Among the EAMG model induction groups, rats in Groups 1, 2, and 3 exhibited higher body weights compared to the other groups, while rats in Groups D and F showed lower body weights. When analyzed separately, the final body weight of most rats in the 50 µg group was around 180 g (Figure 3B), whereas the rats in the 75 µg group showed slower growth, with most remaining below 180 g (Figure 3C). The weight curve for the 50 µg group showed a gradual upward trend, while in the 75 µg groups, weight loss was observed in most groups later in the induction period (Figure 3B,C).

The clinical scores revealed similar trends: the 75 µg group had higher scores, and symptoms appeared earlier than in the 50 µg group (Figure 3D). Specifically, most rats in the 75 µg group began showing typical muscle weakness symptoms around Day 10 (Figure 3F), while some rats in the 50 µg group did not show symptoms until Day 15 or even Day 20 (Figure 3E). Furthermore, the final scores for most rats in the 50 µg group were below 2 points, while some rats in the 75 µg group reached scores above 3 points.

**Figure 4:**
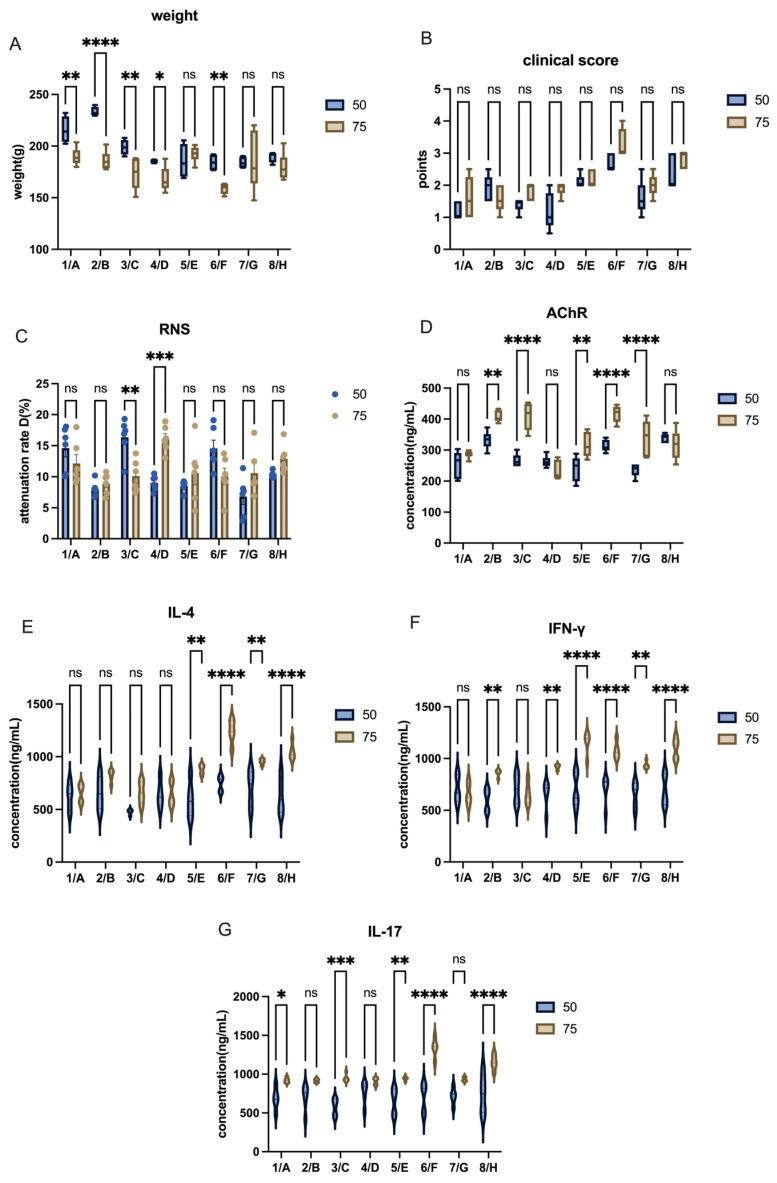
EAMG model evaluation of Lewis rats receiving different doses of AChR 97–116. (**A**) Comparison of body weight after model induction among groups of different AChR doses. (**B**) Comparison of clinical scores among groups receiving different AChR doses. (**C**) Comparison of attenuation rate D value in RNS tests across groups with different AChR doses. (**D**) AChR antibody levels in groups with varying AChR doses. (**E**–**G**) Cytokine levels (IL-4, IFN-γ, IL-17) in groups receiving different AChR doses. All data are expressed as mean ± SEM. Two-way ANOVA was performed between groups. Student *t*-tests were conducted between the two groups. The data are representative of three independent experiments with consistent results (* *p* < 0.05, ** *p* < 0.01, *** *p* < 0.001, **** *p* < 0.0001, ns: not significant).

To compare the effects of different AChR doses, we grouped the animals accordingly. With other conditions kept constant, we compared the body weight and clinical scores at the final stage. As seen in the graph, the 75 µg groups generally showed higher body weights and concentration of AChR antibodies than the 50 µg groups (Figure 4A,D), and pro-inflammatory cytokine levels followed a similar trend (Figure 4E–G). However, the results of the RNS test were unexpected, as only two of the 75 µg groups showed better results than the 50 µg groups (Figure 4C, Table S3). Similarly, the clinical score of different groups showed no significant difference (Figure 4B, Table S4).

**Figure 5:**
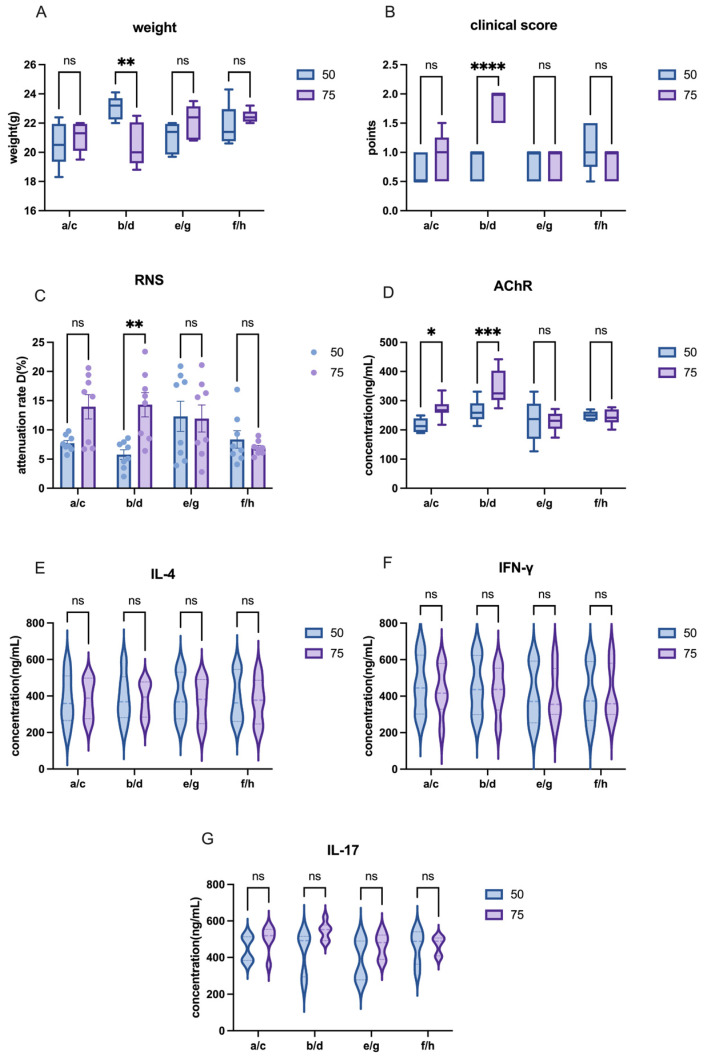
Different doses of AChR influence on EAMG model induction of C57 mice. (**A**) Comparison of body weight among groups receiving different AChR doses. (**B**) Comparison of clinical scores among groups receiving different AChR doses. (**C**) Comparison of attenuation rate D value in RNS tests across groups with different AChR doses. (**D**) AChR antibody levels in groups with varying AChR doses. (**E**–**G**) Cytokine levels (IL-4, IFN-γ, IL-17) in groups receiving different AChR doses. All data are expressed as mean ± SEM. Two-way ANOVA was performed between groups. Student *t*-tests were conducted between the two groups. The data are representative of three independent experiments with consistent results (* *p* < 0.05, ** *p* < 0.01, *** *p* < 0.001, **** *p* < 0.0001, ns: not significant).

In C57 mice, while model induction was unsuccessful, the comparison between different dosages of AChR provided valuable insights. Some of 75 µg AChR group showed higher clinical score and RNS attenuation, accompanied with higher level of AChR level in several groups (Figure 5A–D).

In conclusion, the 75 µg dose of AChR appears to induce a more severe immune reaction, leading to more pronounced MG-like symptoms. Therefore, we decided to apply the 75 µg dose of AChR α 97–116 in subsequent studies.

### 2.3. 2 mg H37Ra Had Better Model Induction Effect

**Figure 6:**
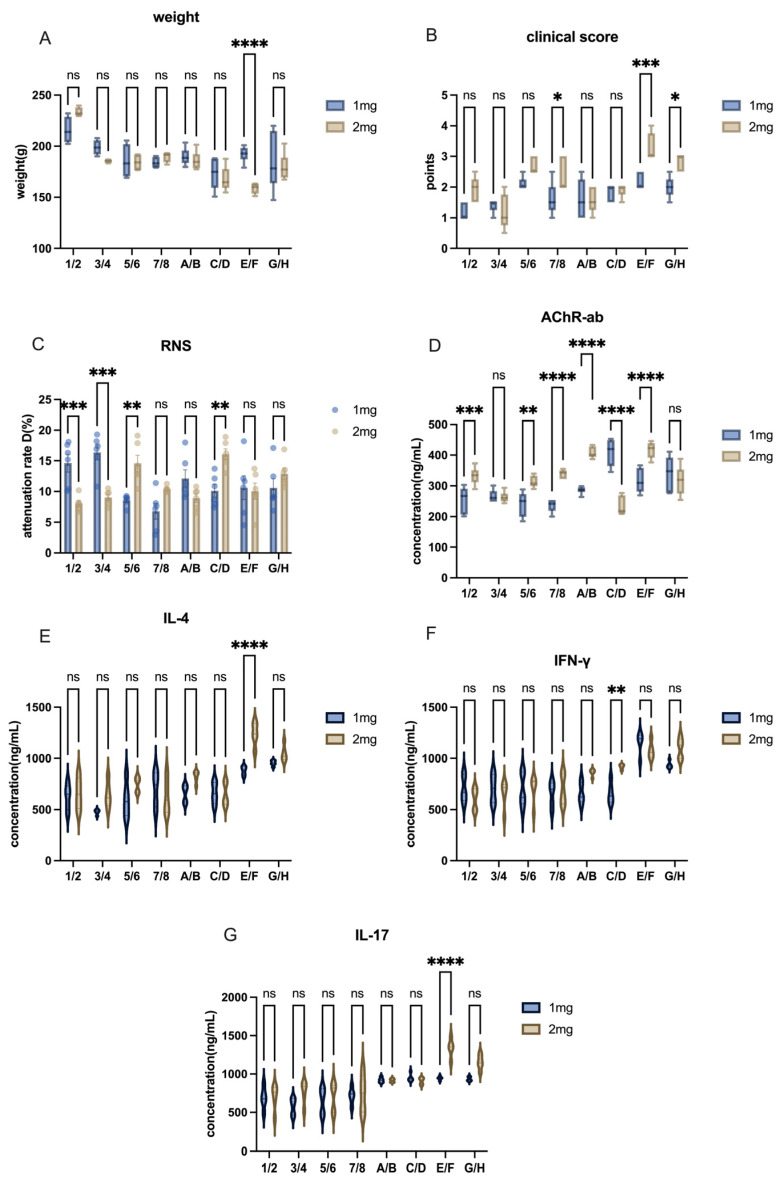
Effects of different doses of H37Ra on Lewis rats. (**A**) Comparison of body weight among groups receiving different doses of H37Ra. (**B**) Comparison of clinical scores among groups receiving different doses of H37Ra. (**C**) Comparison of attenuation rate (D value) in RNS tests across groups with different doses of H37Ra. (**D**) AChR antibody levels in groups receiving varying doses of H37Ra. (**E**–**G**) Cytokine levels (IL-4, IFN-γ, IL-17) in groups administered different doses of H37Ra. All data are expressed as mean ± SEM. Two-way ANOVA was performed between groups. Student *t*-tests were conducted between the two groups. The data are representative of three independent experiments with consistent results (* *p* < 0.05, ** *p* < 0.01, *** *p* < 0.001, **** *p* < 0.0001, ns: not significant).

**Figure 7:**
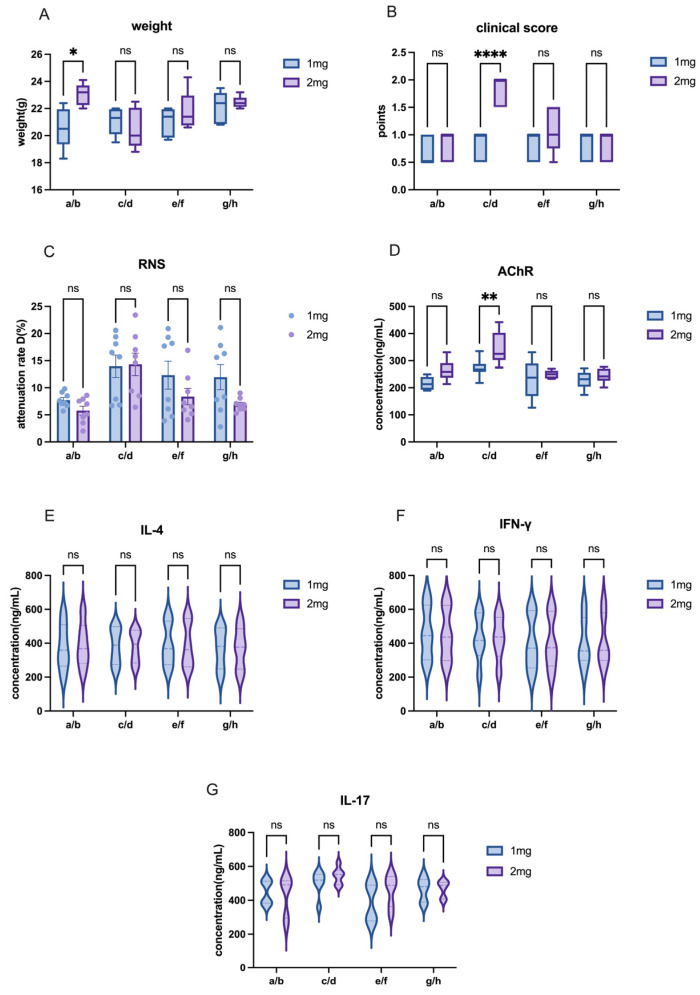
Evaluation of EAMG model in C57 mice with different doses of H37Ra. (**A**) Comparison of body weight among groups receiving different doses of H37Ra. (**B**) Comparison of clinical scores among groups receiving different doses of H37Ra. (**C**) Comparison of attenuation rate D value in RNS tests across groups with different doses of H37Ra. (**D**) AChR antibody levels in groups receiving varying doses of H37Ra. (**E**–**G**) Cytokine levels (IL-4, IFN-γ, IL-17) in groups administered different doses of H37Ra. All data are expressed as mean ± SEM. Two-way ANOVA was performed between groups. Student *t*-tests were conducted between the two groups. The data are representative of three independent experiments with consistent results (* *p* < 0.05, ** *p* < 0.01, **** *p* < 0.0001, ns: not significant).

A comparison between the 1 mg and 2 mg H37Ra groups revealed that, although the differences in weight and inflammatory cytokine levels were not significant across most groups (Figure 6A,E,F), the majority of animals in the 2 mg group exhibited higher concentrations of AChR antibodies (Figure 6D). This suggests a more severe inflammatory response in the 2 mg group. While in C57 mice, there was no significant difference between 1 mg and 2 mg H37Ra (Figure 7).

### 2.4. The Mixing Time Affects Model Induction Efficiency to a Limited Extent

**Figure 8:**
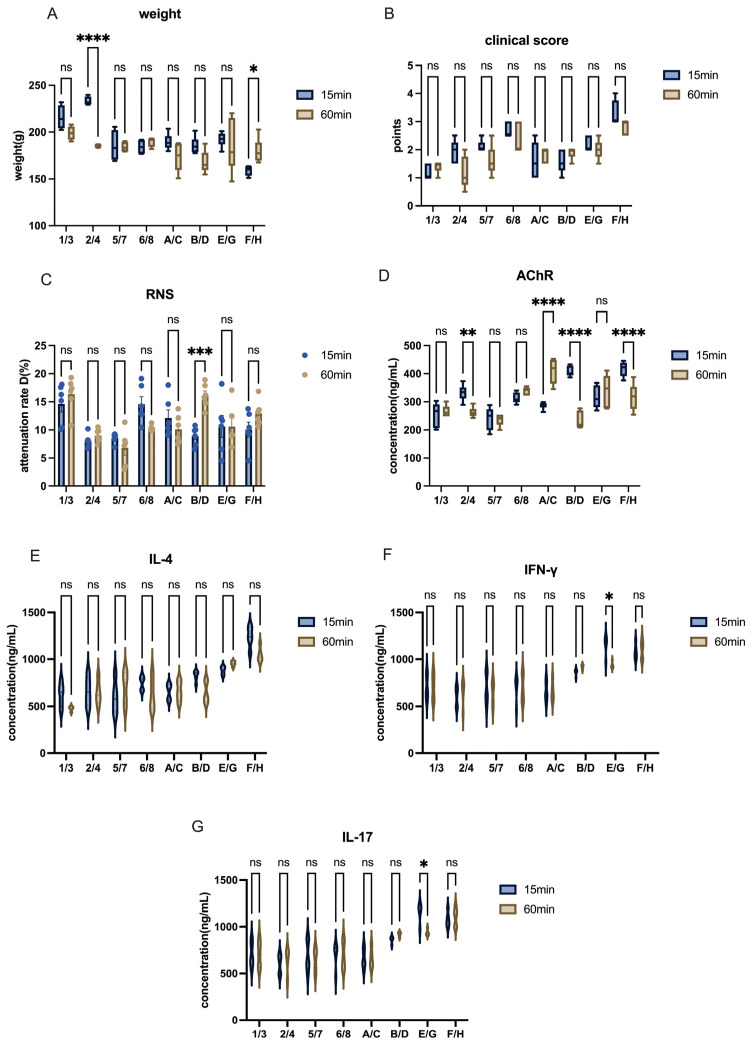
Impact of different mixing times on EAMG model construction in Lewis rats. (**A**) Comparison of body weight among groups with different antigen-adjuvant mixing times. (**B**) Comparison of clinical scores among groups with different mixing times. (**C**) Comparison of attenuation rate D value in RNS tests across groups with different mixing times. (**D**) AChR antibody levels in groups subjected to different mixing times. (**E**–**G**) Cytokine levels (IL-4, IFN-γ, IL-17) in groups with varying mixing times. All data are expressed as mean ± SEM. Two-way ANOVA was performed between groups. Student *t*-tests were conducted between the two groups. The data are representative of three independent experiments with consistent results (* *p* < 0.05, ** *p* < 0.01, *** *p* < 0.001, **** *p* < 0.0001, ns: not significant).

Water and oil formulations require emulsification through repeated agitation to form a stable solution. However, we observed that after the emulsion was injected into the subcutaneous tissue, absorption appeared to be limited, which was often accompanied by the formation of a palpable subcutaneous mass. The mass was firm and encased in a hard capsule, which hindered the absorption of the drug. This led us to hypothesize that excessive emulsification time might have caused the drug to become overly thick, preventing its absorption. To test this hypothesis, we compared the effect of different mixing times on the efficiency of EAMG model induction. The results showed that while varying the mixing time significantly only affected AChR antibodies levels to a certain extent (Figure 8D), no changes were observed in body weight, clinical score evaluation, or level of inflammatory cytokines (Figure 8A,B,E–G). Additionally, the RNS test indicated that the number of EAMG-positive rats in the 15 min mixing time group was not significantly higher than in the 60 min group (Figure 8C).

**Figure 9:**
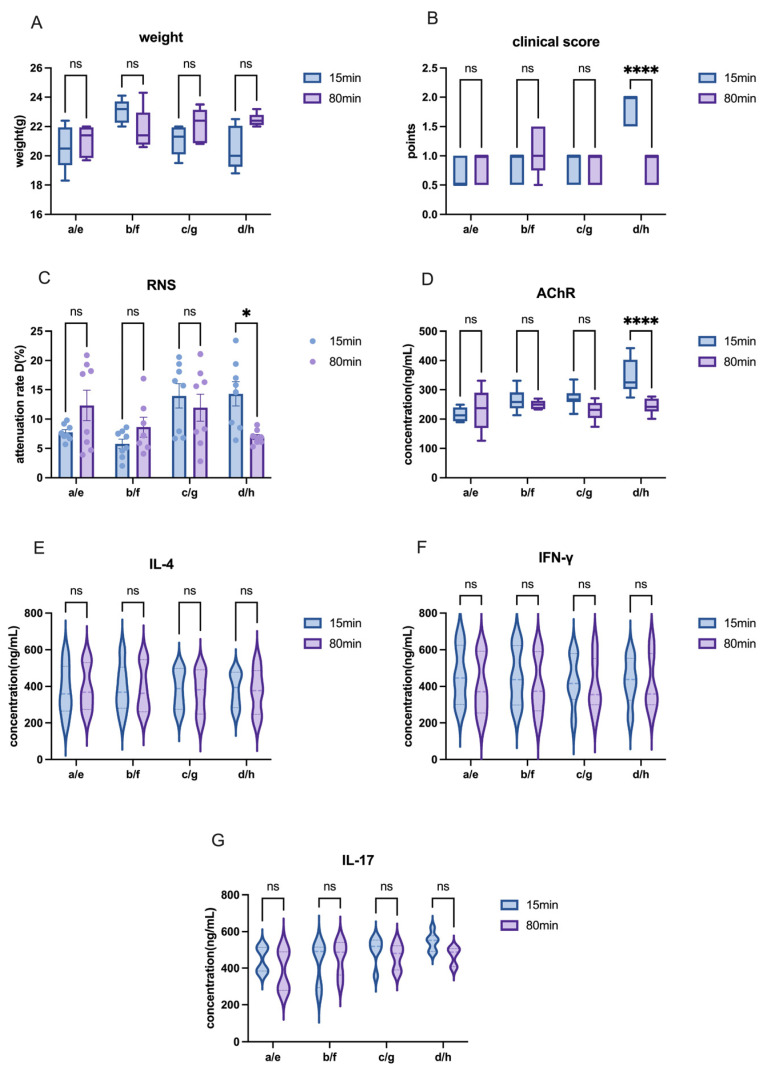
Impact of different mixing times on EAMG model construction in C57 mice. (**A**) Comparison of body weight among groups with different antigen-adjuvant mixing times. (**B**) Comparison of clinical scores among groups with different mixing times. (**C**) Comparison of attenuation rate D value in RNS tests across groups with different mixing times. (**D**) AChR antibody levels in groups subjected to different mixing times. (**E**–**G**) Cytokine levels (IL-4, IFN-γ, IL-17) in groups with varying mixing times. All data are expressed as mean ± SEM. Two-way ANOVA was performed between groups. Student *t*-tests were conducted between the two groups. The data are representative of three independent experiments with consistent results (* *p* < 0.05, **** *p* < 0.0001, ns: not significant).

In C57 mice, we observed that as mixing time increased, the solution became a stable emulsion. Consistent with the observations in Lewis rats, cytokine levels and body weights also showed no significant changes (Figure 9A,E–G). The differences were observed in only one group in RNS and clinical scores, as well as AChR antibodies (Figure 9B–D). The results demonstrated that different mixing times had a minimal effect on the efficiency of model induction. Nonetheless, it may influence drug absorption, with animals in the shorter mixing time groups showing faster absorption rates, with fewer subcutaneous nodules generally observed in animals from the shorter mixing time groups. However, this finding was based on empirical observations and was not formally analyzed statistically.

### 2.5. Foot Pad Injection Could Increase the Success Rate of the Model

**Figure 10:**
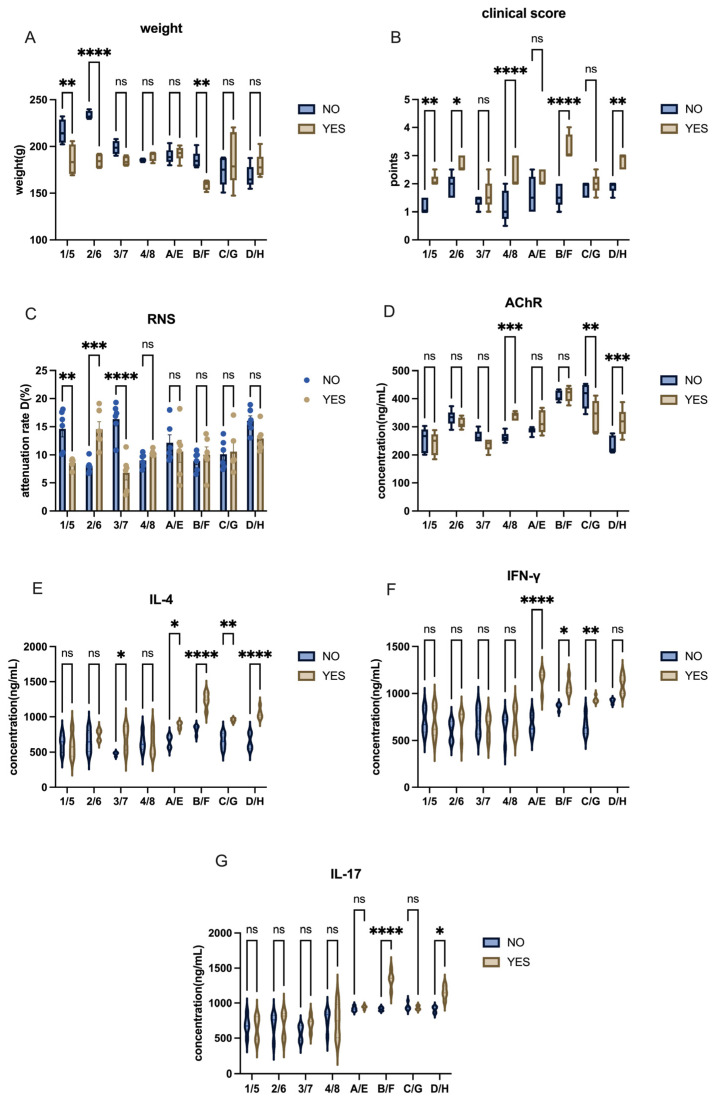
Effects of including or excluding footpad injection on EAMG model induction in Lewis rats. (**A**) Comparison of body weight between groups with or without footpad injections. (**B**) Comparison of clinical scores between groups with or without footpad injections. (**C**) Comparison of attenuation rate D value in RNS tests across groups with or without footpad injections. (**D**) AChR antibody levels in groups with or without footpad injections. (**E**–**G**) Cytokine levels (IL-4, IFN-γ, IL-17) in groups with or without footpad injections. All data are expressed as mean ± SEM. Two-way ANOVA was performed between groups. Student *t*-tests were conducted between the two groups. The data are representative of three independent experiments with consistent results (* *p* < 0.05, ** *p* < 0.01, *** *p* < 0.001, **** *p* < 0.0001, ns: not significant).

The inclusion of footpads as an injection site has been a subject of debate in current experimental protocols. In this study, we investigated the effect of footpad injection on model induction by conducting injections both with and without the inclusion of footpads. The two groups, differing only in the injection site, were compared to assess the outcomes. Rats that received injections into the footpad exhibited symptoms earlier, with a faster onset and more severe manifestation at the final model evaluation, with several footpads injection groups had higher clinical score and attenuation D value, along with lower weight level (Figure 10A–C). The AChR level in specific “include” group were higher than those in the “exclude” group (Figure 10D), accompanied by an elevated level of inflammatory cytokines (Figure 10E–G). In conclusion, footpad injection significantly improved the efficiency of model induction.

## 3. Discussion

In this research, we systematically optimized the induction of the EAMG model in two commonly used animal models: C57BL/6 mice and Lewis rats. Our findings demonstrated that Lewis rats exhibited a significantly higher success rate in model induction compared to C57BL/6 mice, likely due to their genetic predisposition to autoimmune diseases. By refining antigen dosage, adjuvant concentration, and injection techniques, we successfully enhanced the stability and reproducibility of the EAMG model, providing a more reliable platform for future therapeutic investigations. This work offers a systematic comparison of key variables influencing EAMG model induction, including species selection, antigen formulation, injection strategy, and adjuvant use. By establishing an optimized and standardized protocol, our study addresses the current lack of consensus in EAMG model preparation and contributes to improving the reliability, comparability, and translational potential of subsequent investigations.

EAMG has been extensively studied, with initial models relying on TAChR to trigger a robust immune response. However, due to difficulties in obtaining TAChR, most recent studies have adopted synthetic AChR peptides corresponding to the immunogenic region 97–116 of the rat α-subunit [6]. Unlike TAChR, synthetic AChR peptides require multiple high-dose injections combined with immune boosters for effective model induction. Our study confirms this challenge, as the commonly used doses (50 µg AChR + 1 mg H37Ra) were insufficient to induce robust disease symptoms in Lewis rats. By increasing the doses to 75 µg AChR + 2 mg H37Ra, we achieved a more consistent and pronounced autoimmune response.

Despite some studies successfully using recombinant AChR immunization in C57BL/6 mice [12], we encountered significant challenges in model induction in mice. This discrepancy could be attributed to differences in antigen formulation, adjuvant efficacy, or genetic background. In contrast, Lewis rats demonstrated a higher success rate, consistent with their established role in autoimmune disease models such as EAE and EAU. Notably, we chose female animals based on prior evidence that estrogen and X-chromosome-linked immune regulation enhance autoimmune responses [28]. This factor may have contributed to the increased susceptibility of Lewis rats to EAMG in our study.

Through a series of detailed refinements, we improved the efficiency and consistency of EAMG model induction while maintaining immune response stability:

1. Antigen and Adjuvant Dosing: Increasing the concentrations of AChR and H37Ra enhanced disease severity without causing excessive inflammatory cytokine responses.
2. Emulsion Preparation: Proper emulsification was critical for effective antigen delivery. Using syringes without rubber pistons minimized heat generation during mixing and preserved antigen integrity.
3. Injection Technique Optimization: Needle size and injection site were refined to improve delivery. Subcutaneous injections in rats (shoulder/back) were performed using 22 G needles, while 25 G needles were used for mice and footpad injections.
4. Post-Injection Care: Gentle pressure application after injection and regular monitoring reduced subcutaneous nodule formation. Iodine disinfectants and erythromycin ointment were applied when needed to prevent secondary infection.
5. Increased Injection Sites: We observed that distributing the total dose across more injection sites, with reduced volume per site, decreased nodule formation and may enhance absorption. However, this approach requires careful site selection to avoid repeated puncture and injection errors.
6. Massage and Warm Compresses: Post-injection massage and warm compresses appeared to enhance drug absorption and reduce persistent nodules. Animals receiving this care exhibited fewer or smaller nodules at necropsy.

These refinements improved model stability and reproducibility, enabling more consistent clinical phenotypes and providing a reliable platform for therapeutic evaluation.

In the process of EAMG model establishment, several technical factors can influence the efficiency, stability, and reproducibility of disease induction. In this study, we have carefully controlled key technical parameters to minimize variability:

1. The antigen used was a synthetic peptide with 98% purity, which reduces the risk of batch-to-batch variability and ensures consistent immune activation. Peptide purity is known to affect epitope presentation and immunogenicity, and variations in purity could potentially lead to differences in disease severity or immune responses.
2. We specified the adjuvants and immune enhancers, including their commercial sources and formulations, to maintain consistency in immune stimulation. The type and quality of adjuvant can modulate the intensity and character of the immune response, thereby influencing the onset and severity of EAMG symptoms.
3. Injection parameters, such as the volume per site, injection sites, and emulsification conditions, were standardized in our protocol. These factors directly affect antigen distribution and absorption, which in turn may influence the immune response dynamics. For instance, improper emulsification or inconsistent injection volumes could lead to uneven antigen release or local tissue reactions, affecting model consistency.

Despite these controls, we acknowledge that minor variations in these technical factors could still impact the final outcomes of EAMG induction. Therefore, standardizing and reporting these details is crucial for reproducibility across laboratories; however, further inter-laboratory validation will be needed to confirm the generalizability of our protocol.

Noticeably, although C57BL/6 mice exhibited less pronounced clinical manifestations under the conditions tested in this study, their value as a research model should not be overlooked. C57BL/6 mice remain widely used in immunological and genetic research due to the broad availability of transgenic and knockout lines. In specific experimental contexts—particularly those involving genetic manipulation or mechanistic exploration—the use of C57BL/6 mice remains indispensable. Therefore, the selection of an appropriate animal model should be carefully aligned with the specific research objectives and technical requirements. The observed differences in EAMG susceptibility between Lewis rats and C57BL/6 mice may reflect differences in genetic background, immune response characteristics, and overall predisposition to autoimmune diseases, rather than an inherent unsuitability of C57BL/6 mice for EAMG induction.

A major strength of our study is the systematic evaluation of different parameters influencing EAMG model induction, leading to practical improvements in protocol optimization. The modifications we introduced enhance reproducibility while minimizing unnecessary variability in immune response. Additionally, our approach balances immune activation efficiency with reduced trauma to animals, making it suitable for long-term therapeutic studies.

However, some limitations should be acknowledged. First, although ELISA provided valuable insights into inflammatory factor regulation, flow cytometry (FCM) could have offered a more detailed characterization of immune cell populations. The decision to forgo FCM analysis was based on the need to preserve animals for subsequent drug intervention trials, as FCM requires either large blood volumes or splenic immune cell extraction, which would have necessitated animal sacrifice. Future studies should consider non-terminal immune profiling techniques to further validate immune response mechanisms. Second, while our findings demonstrated that higher antigen and adjuvant doses improved model consistency, the long-term impact of dose escalation on immune tolerance remains to be investigated. Third, although consistent clinical outcomes were observed, the relatively small sample size may have limited the statistical power to detect subtle differences. We acknowledge that a priori sample size estimation was not conducted, which is a limitation of this study. Post hoc analysis indicated that a larger sample size would be necessary to achieve adequate statistical power. Due to resource and ethical constraints, we were unable to meet this requirement, which we acknowledge as a methodological shortcoming in our study design.

To conclude, our study provides an optimized strategy for EAMG model construction, particularly in Lewis rats, by refining antigen dose, injection methods, and post-injection care. This study primarily focused on the optimization and standardization of EAMG model induction protocols. These improvements enhance model reproducibility, making it a valuable tool for future research in autoimmune myasthenia gravis. While cellular and molecular mechanisms underlying disease pathogenesis were beyond the scope of the current investigation, we acknowledge that such mechanistic insights are critically important for deepening our understanding of EAMG and advancing the development of targeted therapeutic strategies. Future studies based on the established model are underway to explore pathogenic mechanisms and therapeutic interventions at the cellular and molecular levels, and integration of advanced immune profiling techniques will be essential to elucidate disease mechanisms and identify therapeutic targets.

## 4. Materials and Methods

1. Animals

Female Lewis rats and female C57BL/6 mice were used for the comparative model induction in this study. Both Lewis rats and C57BL/6 mice were purchased from Vital River Laboratory Animal Technology Co., Ltd. (Beijing, China). All animal experimental procedures were approved by the Ethics Committee of Nanjing University of Chinese Medicine on 22 March 2022 (Approval No. 202203A058).

2. Model induction

Lewis rats or C57BL/6 mice all receive 50 µg or 75 µg AChR and 1 mg or 2 mg of H37Ra injection. Precisely, they all received 200 µL inoculum, with 100 µL PBS (contained 50 µg or 75 µg AChR) and 100 µL CFA (contained 1 mg or 2 mg of H37Ra) at first time of immunization. At the second time of immunization (booster immunization), 100 µL PBS (contained 50 µg or 75 µg AChR) and 100 µL IFA (without H37Ra) were instead. The injection method was subcutaneous multi-point injection. The injection sites were the shoulders, the back, and the base of the tail. Rats in specific groups also received injections in the footpad area.

3. Conditions

There were five varieties in this research. The variates were listed in Table 1.

(1) Animals.

The C57BL/6 mice and Lewis rats were both engaged to induce EAMG models.

(2) The dose of AChR α 97–116 peptides.

In previous studies, 50 µg and 75 µg of AChR α 97–116 have been commonly used for EAMG model induction. Most studies have employed the 50 µg dose [29], but some have reported that this amount may not be sufficient to induce a stable immune response, resulting in failure to establish a consistent myasthenic state. Consequently, some researchers have increased the peptide dose to 75 µg [30], aiming to achieve better modeling efficiency.

(3) The dose of H37Ra mycobacterium tuberculosis.

H37Ra mycobacterium tuberculosis, serving as an immune response booster, enhances the immunogenicity of AChR peptides, thereby promoting inflammation and enabling the body to mount a more robust immune response. The majority of model induction protocols employed the dose of 1 mg H37Ra per animal [31]. However, a small number of studies have increased the dose to 2 mg [32], and some have even omitted mycobacterium tuberculosis the during the modeling process [33].

(4) The time to mix the two reagents.

Details such as the method and duration of drug mixing were not explicitly described in the methodology section of previous studies but were instead vaguely referred to as “mixing” in the text.

(5) Injection sites.

Current research has not yet reached a consensus on the optimal injection site. Some studies administered injections in the shoulder and back regions [14], while others extended the injection site to the base of the tail [30]. Additionally, some studies reported injections in the footpad [34].

4. Groups

- C57BL/6 mice

Considering the foot pads of C57BL/6 mice are thin and not suitable for injection, the variables we selected in the mouse experiment were mainly peptides, mycobacterium tuberculosis powder, and emulsification time. The groups of C57BL/6 mice were shown in Table 2.

- C57 mice (comparison between 50 µg AChR and 75 µg AChR)

The other modeling conditions for C57 mice remained consistent, with the only variable being the dose of AChR. In this study, we compared the effects of different AChR doses on model induction by comparing Group a and Group c or Group b and Group d in order to evaluate the impact of varying AChR dosages on model development.

- C57 mice (comparison between 1 mg H37Ra and 2 mg H37Ra)

The other modeling conditions for C57 mice remained constant, with the only variable being the dose of H37Ra Mycobacterium tuberculosis. In this study, adjacent groups were paired to compare the effects of varying bacterial powder dosages on model induction. For instance, Group a and Group b, Group c and Group d, and so on.

- C57 mice (comparison between mixing time of two drugs)

The other modeling conditions of C57 mice remained the same, and the only variable was the mixing time of PBS and CFA or IFA. In this research, each group—separated by one intervening group—was used to detect the effects of different emulsification time on modeling. For example, mixing time in modeling was explained by the comparison between group a and e or between group b and f, etc.

- Lewis rats

The groups of Lewis rats were shown in Table 3.

- Lewis rats (comparison between 50 µg AChR and 75 µg AChR)

The other modeling conditions for Lewis rats remained consistent, with the only variable being the dose of AChR. In this study, we compared the effects of different AChR doses on model induction by using Groups 1–8 (50 µg AChR) and Groups A–H (75 µg AChR) in order to evaluate the impact of varying AChR dosages on model development.

- Lewis rats (comparison between 1 mg H37Ra and 2 mg H37Ra)

The other modeling conditions for Lewis rats remained constant, with the only variable being the dose of H37Ra Mycobacterium tuberculosis. In this study, adjacent groups were paired to compare the effects of varying bacterial powder dosages on model induction. For instance, Group 1 and Group 2, Group A and Group B, and so on.

- Lewis rats (comparison between mixing time of two drugs)

The other modeling conditions of Lewis rats remained the same, and the only variable was the mixing time of PBS and CFA or IFA. In this research, each group—separated by one intervening group—was used to detect the effects of different emulsification times on modeling. For example, mixing time in modeling was explained by the comparison between group1 and group3 or group A and group C, and etc.

- Lewis rats (comparison between injection sites)

The other modeling conditions for Lewis rats remained consistent, with the only variable being the inclusion or exclusion of footpad injection sites. In this study, four groups were used to assess the effects of footpad injections on model induction. For example, the role of footpad injection was examined by comparing Group 1 and Group 5, or Group A and Group E.

5. Materials

According to Baggi’s methods [8], a synthetic R97–116 peptide (DGDFAIVKFTKVLLDYTGHI) was employed to induce EAMG rats, corresponding to the rat AChR-α subunit. The peptide, obtained from ChinaPeptides Co., Ltd. (Wuhan, China), underwent rigorous quality control procedures and had a purity of 98%. CFA and IFA, adjuvants for injection, were bought from Sigma-Aldrich (St. Louis, MO, USA). The 1 mL, 2 mL, and 5 mL injectors with Mycobacterium tuberculosis (strain H37RA) were all obtained from BD bioscience (Franklin Lakes, NJ, USA). In the repetitive nerve stimulation (RNS) test, the bioelectrical signal transduction system (Cat. BL-420N; Tai Meng, Chengdu, China) was used to examine the muscle function. Acupuncture needles came from Huatuo Pharmaceutical (Zhengzhou, Henan, China). Reagents related to the ELISA test were provided by Jin Yibai Biotechnology Co., Ltd. (Nanjing, China).

6. Body weight

Body weight was measured and taken at the same time every four days by a double-blind method. The weight curve graph was generated using Prism 10.

7. Clinical score evaluation

The clinical scores of all animals were recorded from ay 1 to Day 45 in a double-blind manner, referred to as LA Vennon. When scoring, animals should be placed on a smooth surface and observed continuously for about two minutes. Researchers then asses the animal’s mobility against the scoring criteria and assign the grade that best matches its behavior. The clinical score criteria are shown in Table 4.

8. Affinity detection of anti-AChR 97–116 antibody

Referring to previous research methods [35], at Day 45, whole blood was collected from the tail tips of C57 mice or Lewis rats. After allowing the samples to stand for 30 min at room temperature (RT), they were centrifuged at 3000 rpm for 15 min at 4 °C, and the serum was stored at −20 °C for future experiments. The relative affinity of anti-AChR97–116 antibodies in the serum was evaluated using enzyme-linked immunosorbent assay (ELISA). Following the kit instructions, 96-well flat-bottom plates from Corning Incorporated (Corning, NY, USA) were coated with AChR97–116 peptide (5 μg/mL in 100 μL) and incubated overnight at 4 °C. The plates were then washed with PBS-T (0.05% PBS in Tween 20) the next day and blocked with 250 μL of 10% fetal calf serum (FCS) at RT for 2 h. Serum samples (diluted 1:5000) were added in a total volume of 100 μL and incubated at RT for 2 h. After washing, biotinylated rabbit anti-rat IgG (1:2000; Boosen Biology, Beijing, China) was added and incubated for 1 h at RT, followed by another washing step. Streptavidin–horseradish peroxidase (1:1000; Jackson, Philadelphia, PA, USA) was then added and incubated at RT for 30 min. After the final wash, tetramethylbenzidine (TMB) substrate solution was added, and the reaction was allowed to develop at RT in the dark.

9. Repetitive Nerve Stimulation (RNS)

After model induction, at Day 45, all animals would undergo the RNS test to assess muscle function after repeated low-frequency electrical stimulation of EAMG rats. Using the methodology of a previous study, four acupuncture needles served as electrodes to connect the muscle to the stimulator. These electrodes were inserted into four distinct regions of each rat: the muscle near the sciatic nerve, abdominal subcutaneous tissue, achilles tendon, and the medial head of the gastrocnemius muscle, which served as the stimulating electrode, reference electrode, and two recording electrodes, respectively. Electrical stimulation was applied in three groups, each consisting of ten pulses at frequencies of 3 Hz and 5 Hz. Each mouse or rat underwent three sets of tests. The attenuation rate (D) was calculated by comparing the first and fifth spikes, using the formula: D = [(first spike value − fifth spike value)/first spike value] × 100%. A D value greater than 10% indicated positive results, suggesting muscle strength impairment.

10. Determination of cytokine concentration

Pro-inflammatory cytokine levels of IL-4, IFN-γ, and IL-17 were measured using ELISA kits provided by Jin Yibai Biotechnology Co., Ltd. (Nanjing, China) to assess the hyperinflammatory state in EAMG animals. The method for collecting whole blood samples from the tail tips and obtaining serum were the same as described in previous section. Serum samples were diluted 4-fold according to the instructions of the kit. A 96-well plate was coated overnight at 4 °C with 100 µL of specific capture antibody (provided by the kit). After incubation, the plate was washed with PBS buffer to remove unbound antibodies. To reduce non-specific binding, 200 µL of 5% bovine serum albumin (BSA) solution was added to each well and incubated at RT for 1 h, followed by washing with PBS. Standard samples and serum samples (100 µL each) were added to the wells and incubated for 2 h to allow the cytokines to bind to the capture antibody. Then, the plate was washed 4 times with PBS to remove unbound samples and 100 µL of HRP-conjugated detection antibody (provided by the kit) was added to each well and incubated for 1 h at RT. After another 4 rounds of PBS washing, 100 µL of TMB substrate solution was added to each well, and the plate was incubated for 10–30 min at RT until a significant color change occurred. The reaction was stopped by adding 100 µL of stop solution. Absorbance was measured at 450 nm using a microplate reader (EnVision, PerkinElmer), and cytokine concentrations were calculated by comparing the optical density (OD) values of the samples with the standard curve generated from known concentrations of the standards.

11. EAMG model evaluation

In this research, we classified mice or rats with clinical score > 2 points and RNS attenuation rate > 10% as successful EAMG models. Meanwhile, the concentration of AChR-ab antibodies and the body inflammation level were used as reference indicators. The high expression of AChR-ab in vivo and high levels of inflammatory cytokines were required in EAMG models as well.

12. Statistical analysis

SPSS 26.0 and GraphPad Prism 10 are used for statistical analysis. The data were presented as mean ± SEM. Comparison between two groups were conducted by Student *t*-tests. Comparisons among multiple groups were carried out using one-way and two-way analysis of variance (ANOVA) (*p*-values < 0.05 indicated statistical significance).

## 5. Conclusions

Conclusively, we reported the optimized method of EAMG induction in this research, confirming the optimal dosage and injection details of the drug. Meanwhile, we evaluated the induction of EAMG model in less invasive but more effective ways, which might provide a foundation for the further study of in-depth aspects of MG.

## Figures and Tables

**Table 1:** Different variates of research.

Variates		
Animals	Lewis rats	C57BL/6 mice
AChR peptides	50 µg	75 µg
H37Ra	1 mg	2 mg
Emulsification time	10–20 min	>1 h
Injection sites	Shoulders and backs	Shoulders, backs, and foot pads

**Table 2:** Group of C57BL/6 mice (n = 8).

Groups	AChR (µg)	H37Ra (mg)	Time (min)	Remark
Control (con)	/	/	/	No operation
Adjuvant (ad)	/	/	20	Inject mixture of PBS and CFA or IFA
a	50	1	15	
b	50	2	15	
c	75	1	15	
d	75	2	15	
e	50	1	80	
f	50	2	80	
g	75	1	80	
h	75	2	80	

**Table 3:** Group of Lewis rats (n = 6).

Groups	AChR (µg)	H37Ra (mg)	Time (min)	Footpads (Y/N)	Remark
Control (CON)	/	/	/	/	No operation
Adjuvant (AD)	/	/	20	N	Inject mixture of PBS and CFA or IFA
1	50	1	15	N	
2	50	2	15	N	
3	50	1	60	N	
4	50	2	60	N	
5	50	1	15	Y	
6	50	2	15	Y	
7	50	1	60	Y	
8	50	2	60	Y	
A	75	1	15	N	
B	75	2	15	N	
C	75	1	60	N	
D	75	2	60	N	
E	75	1	15	Y	
F	75	2	15	Y	
G	75	1	60	Y	
H	75	2	60	Y	

**Table 4:** Lennon clinical score.

Clinical Score ^1^	Symptoms
Grade 0	No muscle weakness
Grade 1	Mildly decreased of activity
Grade 2	Presence of clinical signs after exercise
Grade 3	Severe clinical signs
Grade 4	Death

^1^ If the clinical manifestations are between the two levels, 0.5 point was added.

## Data Availability

The original contributions presented in this study are included in the article and Supplementary Materials. Further inquiries can be directed to the corresponding authors.

## Supplementary Materials

The following supporting information can be downloaded at: https://www.mdpi.com/article/10.3390/ijms26104628/s1.

## Abbreviations

The following abbreviations are used in this manuscript:

MG	myasthenia gravis
EAMG	experimental autoimmune myasthenia gravis s
CFA	complete Freund’s adjuvant
NMJ	neuromuscular junction
AChR	acetylcholine receptor
IFA	incomplete Freund’s adjuvant
MuSK	muscle-specific tyrosine kinase
IL	interleukin
